# Edge contact dependent spin transport for n-type doping zigzag-graphene with asymmetric edge hydrogenation

**DOI:** 10.1038/srep04038

**Published:** 2014-02-10

**Authors:** Xiaoqing Deng, Zhenhua Zhang, Guiping Tang, Zhiqiang Fan, Huali Zhu, Changhu Yang

**Affiliations:** 1 School of Physics and Electronic Science, Changsha University of Science and Technology, Changsha 410114, China

## Abstract

Spin transport features of the n-type doping zigzag graphene nanoribbons (ZGNRs) with an edge contact are investigated by first principle methods, where ZGNRs are C–H_2_ bonded at one edge while C–H bonded at the other to form an asymmetric edge hydrogenation. The results show that a perfect spin filtering effect (100%) in such ZGNR nanojunctions can be achieved in a very large bias region for the unchanged spin states regardless of bias polarities, and the nanojunction with a contact of two C–H_2_ bonded edges has larger spin polarized current than that with a contact of two C–H bonded edges. The transmission pathways and the projected density of states (PDOS) demonstrate that the edge of C-H_2_ bonds play a crucial role for the spin magnetism and spin-dependent transport properties. Moreover, the negative differential resistance (NDR) effect is also observed in the spin-polarized current.

Spintronics is a popular topic in condensed matter physics, materials chemistry, and electronics industry, and the search for suitable spintronic materials is a basic but crucial task^1^,^2^,^3^. At present, we are witnessing intensive investigations on zigzag-edge graphene nanoribbons (ZGNRs) due to their potential applications in spintronics, including spin-polarized current rectification^4^, switching^5^, spin filtering effects^6^, giant magnetoresistance phenomenon^7^, etc. However, the ground state of ideal ZGNRs with mono-hydrogen terminations is found to be antiferromagnetic (AFM)^8^. The spin is parallel at each zigzag edge but antiparallel between the two edges with zero total magnetic moment. Currently, The ZGNRs can be transformed to half-metals by edge modification^9^, applying external electric field^10^, doping other atoms^11^ and topologic line defects^12^, to break the spin degeneracy. In order to stabilize the edges of the GNRs, the dangling bonds of the edge carbon atoms have to be passivated by various ways, including symmetric monohydrogenation ((H–ZGNR–H)^13^,^14^, symmetric dehydrogenation (H_2_–ZGNR–H_2_)^15^,^16^, and asymmetric hydrogenated ZGNR (H_2_–ZGNR–H)^17^,^18^, which are C–H_2_ bonded at one edge while C–H bonded at the another. Recently, through studies on the chemical potential of hydrogen, researchers demonstrated that the composition of sp^2^ and sp3 types at the edges of the GNRs can be easily controlled which indicates that the monohydrogen-terminated and dihydrogen-terminated grapheme nanoribbon heterojunction can be fabricated with experiment via temperature and pressure of H_2_ gas^19^,^20^,^21^. This grapheme nanoribbon heterojunction can be observed by scanning tunnelling microscopy (STM) images (experiment)^21^. Kang. et al. found that the DOS distribution of H_2_–ZGNR–H has two peaks near E_F_^22^. The valence states just below E_F_ belong to the up spin whereas the conduction states just above E_F_ correspond to the down spin. It is worth to note that spin-polarized electronic states localized on the two edges in ZGNRs are individually ferromagnetically (FM) ordered but AFM coupled to each other through the graphene backbone^23^, and that the current mostly flows along the edge of GNR under low bias^24^. As we know, the spin-polarized current is normally produced using “half-metallic” materials, and it is also affected by the magnetic configurations, for example, a symmetric ZGNR plays dual spin diode effect under finite bias with [1,−1] magnetic configuration, but this effect disappear with [1, 1] magnetic configuration, showing symmetric current-voltage characteristics^4^. The [1, 1] magnetic configuration may be more advantageous than [1,−1] because the central part of devices is very small. On the other hand, the range of bias for the spin-filtering effect is an important technique parameter for a spintronics device. However, there is only a narrower bias range for the spin filter in current designed devices. For example, Zeng, et al^15^ presented a dual spin diode with the M-ZGNR-H/N-ZGNR-H2 structure, but its bias range for the spin filter is less than 0.5 V, and the other studies showed that the bias region for perfect spin-filtering is less than 0.2 V in the ZGNR-H/ZGNR-O heterostructure^6^ and carbon atomic chains^25^. Therefore, it is highly necessary to design some new structures to increase the bias range for perfect spin-filtering.

In this present work, based on the H_2_–6ZGNR–H structure, an interesting bipolar magnetic semiconductor whose top valence bands and bottom conduction bands have opposite spin orientations in the proximity of the Fermi level, we investigate its nitrogen-doping effects and find that it can be tuned to a half-metal only with the β-spin conducting channel crossing the Fermi energy after doping. The studies on edge contact configurations show that a perfect spin filtering effect (100%) can be achieved with the unchanged spin states in a very large bias region from −0.5 V to 0.5 V without the magnetic field being applied.

## Results

**Figure 1:**
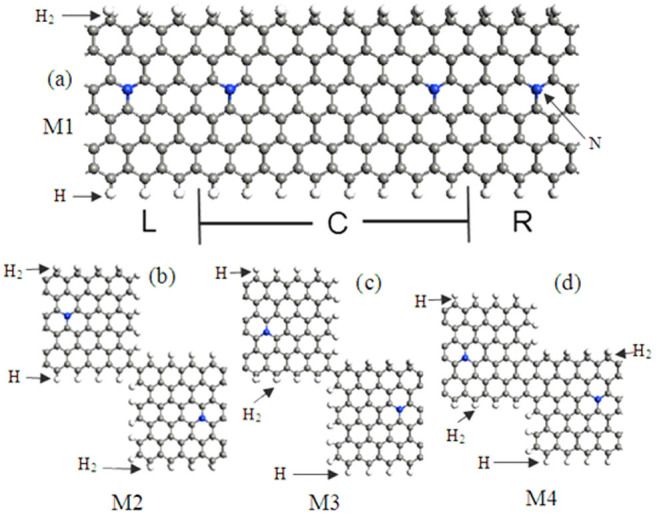
The geometric structures of M1 ∼ M4. where L, R, and C indicate the left and right electrodes, and the central scattering region, respectively, the blue spheres denote doping N atoms, the gray (white) spheres denote C(H) atoms.

In our models as shown in figure 1, the ribbon width of the H_2_–ZGNR–H is characterized by the number of zigzag-shaped C chains, N_z_, along the direction perpendicular to the nanoribbon axis, and the H_2_–ZGNR–H with N_z_ = 6, namely H_2_–6ZGNR–H, is taken as a example. As we know, spin-polarized electronic states mainly localize at the two edges of ZGNRs. While H_2_–6ZGNR–H has two different edge structures: C–H_2_ bonded edge and C–H bonded edge, respectively. This means that there exist different spin-polarized situations at two edges of H_2_–6ZGNR–H. To investigate the spin-polarized current properties along the different edges of H_2_–6ZGNR –H, we design four distinctive devices: M1–M4. In M1, the electron transmission occurs from one electrode to another just along the identical two edges, respectively. But for M2 (M3), we only allow C–H (C–H_2_) edge to transmit the electron between two electrodes. As a comparison, we design M4 on the basis of M3, where the electrons can transmit through C–H_2_ edge and three lays carbon atoms. These devices are shown in figure 1 (a) ∼ (d), respectively. Each device is composed of the left electrode, scattering region (the device region), and right electrode, marked by L, C, and R, respectively. As a semiconductor, the H_2_–6ZGNR–H would feature a very small current under bias, which is unable to perform good comparative studies among these devices. Therefore, we dope these devices with nitrogen (N) atom to increase the electrical conductivity^26^, namely, one carbon atom in the electrode cell is substituted by one N atom.

To find structural stability of asymmetric H_2_-ZGNR-H formed from symmetric H-ZGNR-H, the formation energy can be calculated by the following definition 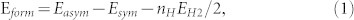
Where *E_asym_* and *E_sym_* are the total energies of asymmetric ZGNRs with sp^2^-sp^3^ edges and symmetric ZGNRs with both sp^2^ edges, respectively. *n_H_* is the number difference of H atoms between the two systems, and *E_H_*_2_ is the energy of an isolated H_2_ molecule. Our calculations show that E*_form_* is −1.2 eV per unit-cell for the H_2_–6ZGNR–H, the negative value of E*_form_* implies that the formation of asymmetric edge is an exothermic reaction from the symmetric 6ZGNRs and H_2_,and that the asymmetric H_2_–6ZGNR–H are energetically more favorable than the symmetric H–6ZGNR–H regardless of the ribbon width. We examine the magnetic properties of H2–6ZGNR–H and consider the following spin configurations: NM, FM and AFM state. When the temperature is 300 K, the FM state is ground state and its total energy is 20 meV per unit cell lower than that of AFM state, 80 meV lower than the nonmagnetic state. When the temperature increase 600 K, its total energy of the FM state is 18 meV per unit cell lower than that of AFM state, 60 meV lower than the nonmagnetic state, and the FM state is ground state. We also compare the results between Generalized Gradient Approximation (GGA) exchange-correlation functional with the above local spin density approximation (LDA or LSDA). At 300 K with GGA (SGGA), the FM state is ground state and its total energy is 30 meV per unit cell lower than that of AFM state, 140 meV lower than the nonmagnetic state which also indicating the FM state is ground state. For the energy bands of unit cells and the electron transmission of devices, we also find the results are consistent qualitatively, therefore, we will not list the dates for the length limitation of papers.

**Figure 2:**
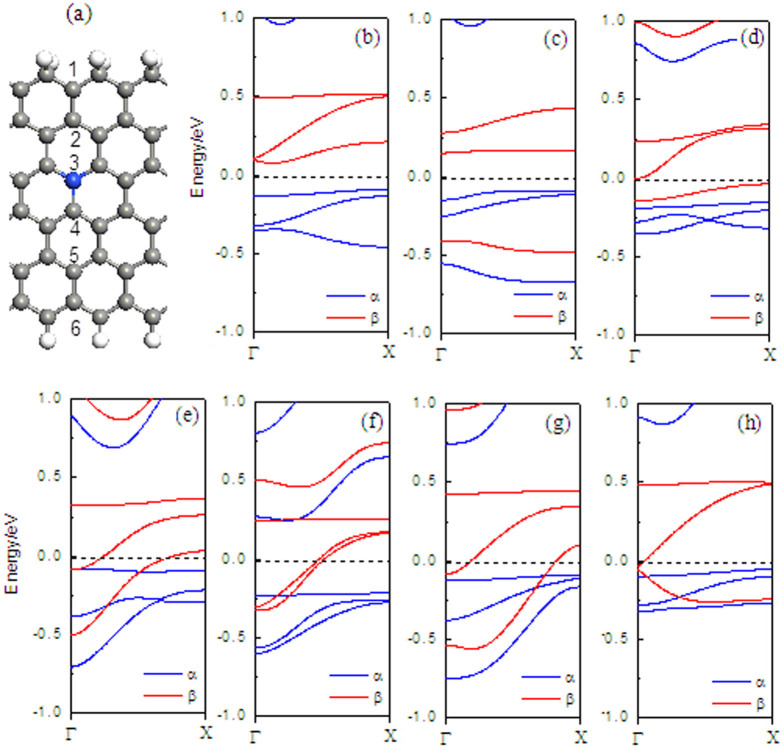
Supercell of H_2_-6ZGNR-H. (a) Supercell of H2-6ZGNR-H doped with N atom, in which three unit cells along the ribbon are included, (b) the band structures of ZGNR without doping N. (c) ∼ (h) with N-doping at Pi (i = 1 ∼ 6), The Fermi level is set to zero throughout the paper.

**Figure 3:**
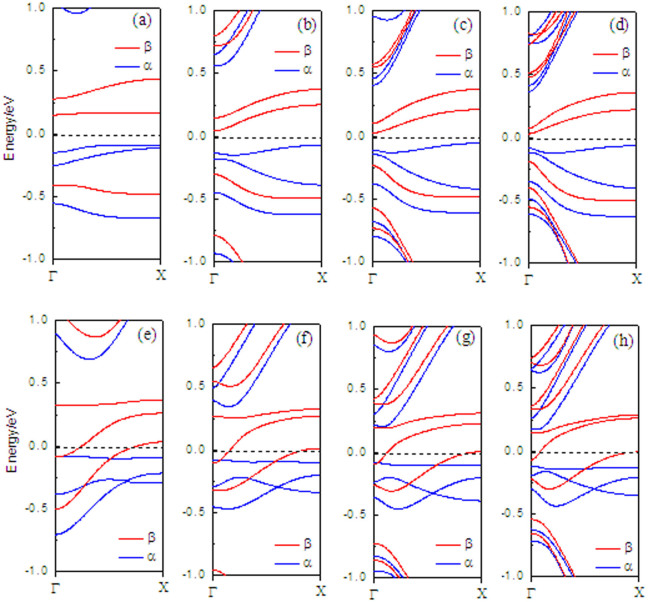
Band structures with N-doping at P1 for (a) 6-ZGNR (b) 10-ZGNR; (c) 14-ZGNR; (d) 18-ZGNR, and with N-doping at P 3 for (e) 6-ZGNR (f) 10-ZGNR; (g) 14-ZGNR; (h) 18-ZGNR.

**Figure 4:**
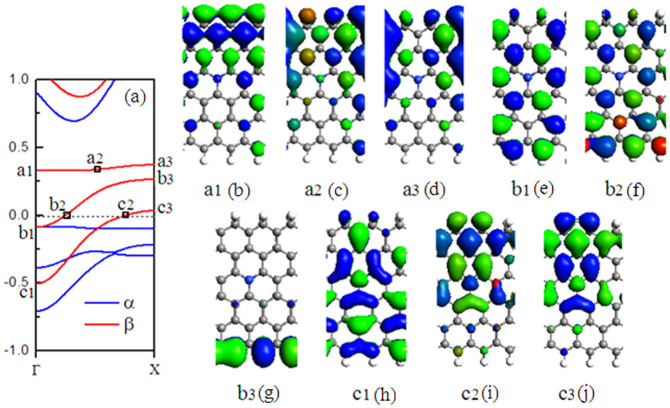
Band structures with N atom doped at P3. (a) band; (e) to (m) exhibit the wave functions corresponding to each points of a1 ∼ c3 in the (a).

In the following, we investigate the band structures for N-doping models, mainly focusing on the doping-position effects on the electronic structures of the H2–ZGNR–H with the distance from the N atom to the ribbon edges. The conductivity of the doped ribbon is highly dependent on the doping site as can be seen from the band structures. When one edge carbon atoms (at P1) is substituted by N atoms, the ribbon is semiconducting (see figure 2c), which is like the band structure without doping (see figure 2b), the bottom conduction band is related to the β-spin state. On the other hand, for the α-spin, the band below the Fermi level serves as the top valence band. When the N atom is doped at Pi (i = 2 ∼ 6), the bottom conduction band is the β-spin bands, which cross the Fermi level. Therefore, the nanoribbon is transformed to a half-metallicity. This is because the dopant atom N introduces an electron to the nanoribbon, which makes the Fermi level shift up to the conduction bands and cross with the conduction bands. When the N atom is doped at P3, P 4, and P5, two β-spin bands cross the Fermi level (see figures 2e–2g). In order to investigate the robustness of doping scheme H2-ZGNR-H with different ribbon widths, calculations are repeatedly performed with varying ZGNR width (6-ZGNR, 10-ZGNR, 14-ZGNR, and 18-ZGNR), as shown in figure 3. Similar results are obtained. In each case, when N is doped at P1 and P3, semiconducting behaviors and half-metallicity can always be observed irrespective of the ribbon width. The only difference is that the band gap of the insulating channel decreases inversely with the increase of the ribbon width. Then, we investigate the nature of the edge states in the bands when the N atom is doped at P3, as shown in figure 4: a1 to c3 are some point at three β-spin bands near the Fermi energy, and (b) to (j) exhibit the wave functions of orbitals corresponding to each point with β-spin. It can be seen that the wave function at b3 is localized at the C–H edge, and the wave functions at a1, a2, a3, c2, and c3 are all localized at the C–H_2_ edge, the others are distributed in the whole supercell.

**Figure 5:**
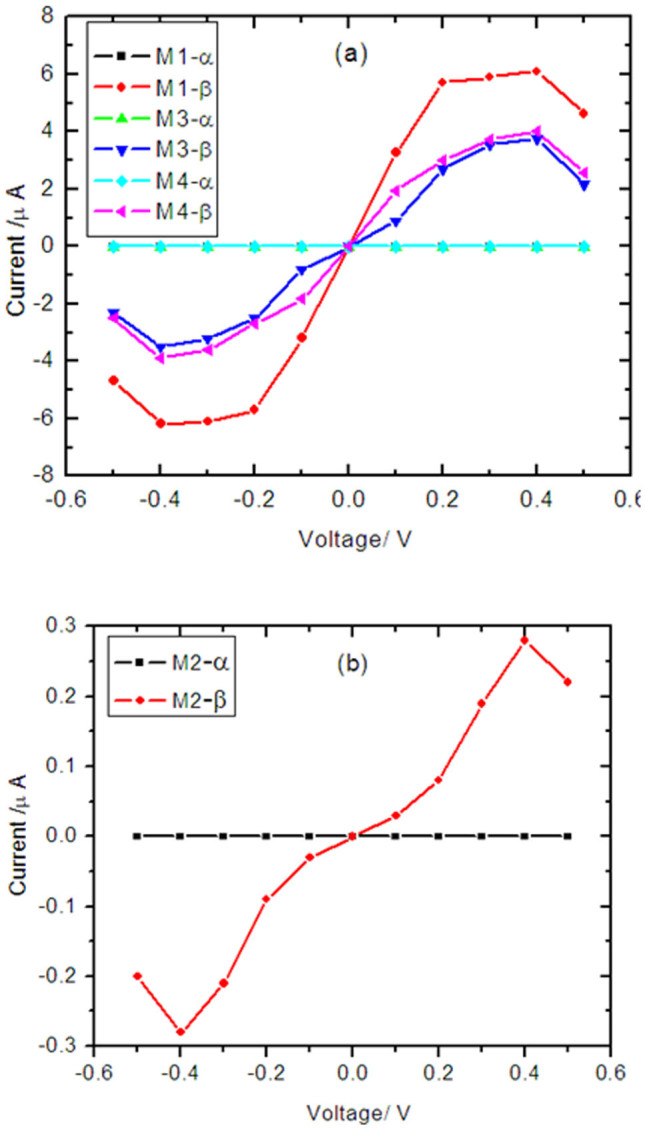
The I – V curves for the α-spin and the β-spin states at various bias, (a) for M1, M3, andM4, and (b) for M2.

**Figure 6:**
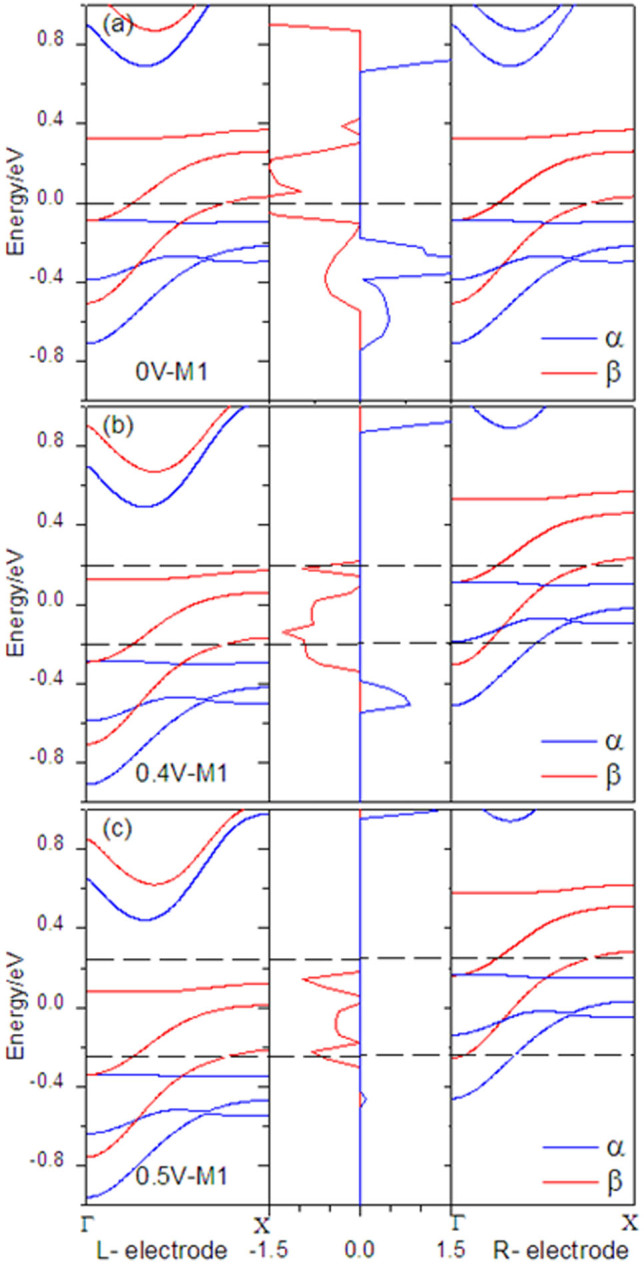
Band structure for the left electrode, transmission spectrum, and band structure for the right electrode, for M1 at 0, 0.4, and 0.5 V bias. The dashed lines denote the chemical potentials of left and right electrodes, and the Fermi level is set to zero.

Next, we investigated the edge contact effects on the spin transport for four models with doping N atom. Figure 5a displays the current as a function of applied bias for models M1, M3, and M4, and figure 4b shows that for M2 separately. The current *I_σ_* through a nanojunction can be calculated from the Landauer-like formula^27^: 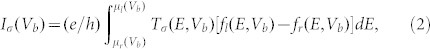
where *V_b_* is the external bias, σ = ↑ (spin up) and ↓ (spin down), *T_σ_*(*E*,*V_b_*) is the bias-dependent transmission coefficient, *f_l_*_/*r*_(*E*,*V_b_*) is the Fermi–Dirac distribution function of the left (right) electrode, As a result, the electrochemical potentials correspond to *μ_l_*(*V_b_*) = *μ_l_*(0) − *eV_b_*/2 and *μ_r_*(*V_b_*) = *μ_r_*(0) − *eV_b_*/2, when the external bias is *V_b_*. Consider the fact that the Fermi level is set to be zero, the region of the energy integral window [*μ_l_*(*V_b_*),*μ_r_*(*V_b_*)] can be written as [−*V_b_*/2, *V_b_*/2]. As can be seen, the current for α - spin state are completely suppressed, while the current for β - spin component is obvious lager than that for the α-spin state. The corresponding spin polarization defined as| (I _α_ − I _β_)/(I _α_ + I _β_) reaches 100% regardless of bias polarities, which shows a perfect spin filter effect in a very large bias region. More importantly, the current of M2 shows one order of magnitude smaller than that of the other models. An interesting negative differential resistance (NDR)^3^,^28^ phenomenon is also observed in these systems, with an occurrence of the maximum current at ±0.4 V. In order to understand the spin filter effect and NDR effect of currents in our case, we chose M1 as the representative. The band structure for the left electrode, transmission spectrum, and band structure for the right electrode at a series of biases 0.0, 0.4, and 0.5 V are plotted in figure 6, which is the most intuitive representation of quantum transport behaviors of a device. Not only the shapes of the transmission spectrum but also the location of transmission peak changes with bias voltages, there are two high and broad β-spin transmission peaks occurring near Fermi energy under zero bias. When the bias is increased to 0.4 V or 0.5 V, the chemical potentials of left (right) electrodes further shift upward (downward) within the bias window, only the β-spin band of the left and right electrodes have an overlap within the bias window, the β-spin electron transmission from left electrode to right electrode is allowable, so only β-spin currents emerge, and the α - spin currents is zero. When the negative bias is applied, the chemical potentials of left (right) electrodes further shift upward (downward), there is no α - spin band overlaps and thus no α-spin current occurs within the bias window. At 0.4 V and 0.5 V bias, the bias window is [−0.2, 0.2] V and [−0.25, 0.25] V, the transmission coeffcients within bias window at 0.4 V are larger than that at 0.5 V. The current is the integration of transmission coeffcients in the bias window according to Landauer-Büttiker formula, thereby, the current at 0.4 V is larger than that at 0.5 V, leading to the NDR phenomenon.

**Figure 7:**
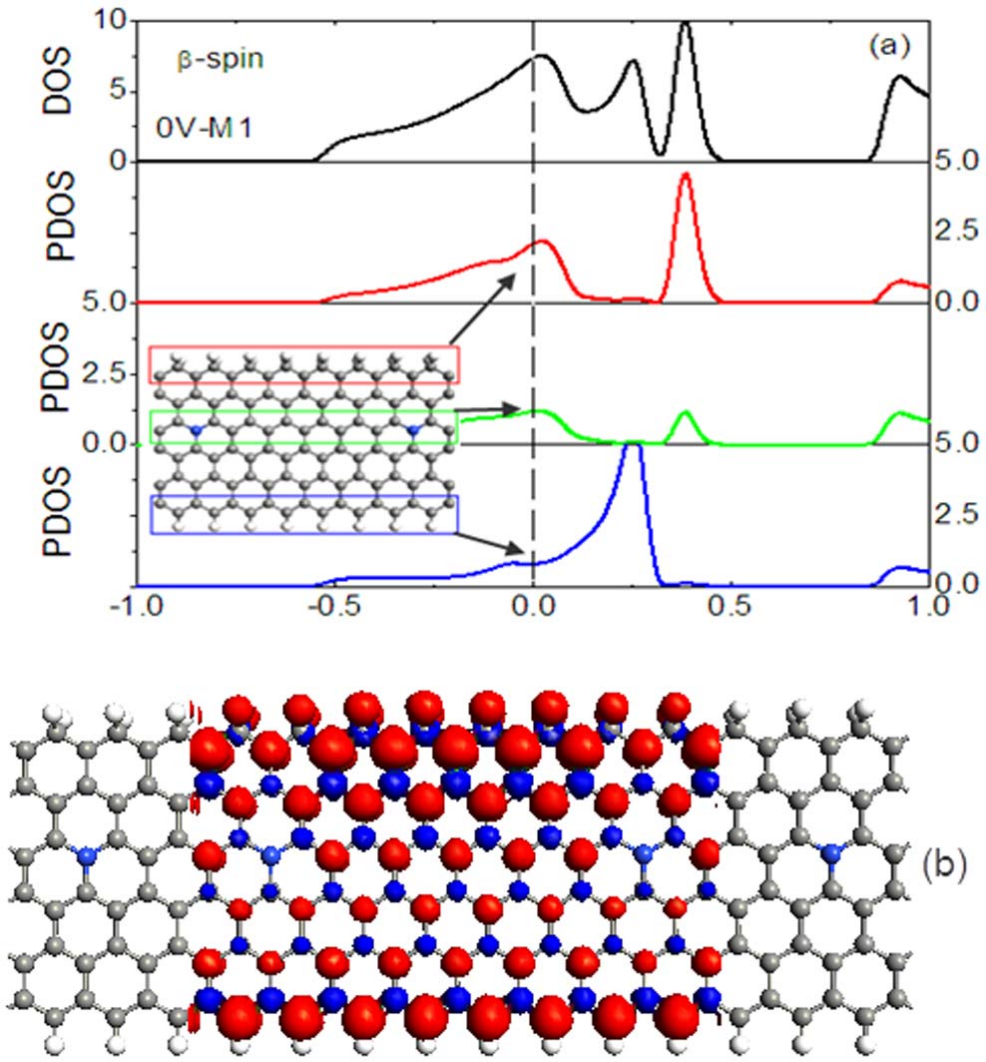
The DOS, PDOS, and spin density. (a) The DOS and PDOS, (b) he isosurface of spin density (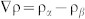)for M1, where the red and blue colors stand for the β-spin and α-spin components, respectively, and the isosurface level is taken as ±0.01|e|/Å^3^.

Now, we take the β- spin state of M1 as an example to understand edge contact effects, the device density of states (DOS) and the projected density of states (PDOS) at zero bias are calculated, as depicted in figure 7a. The PDOS here is the density of states projected on three parts: C and H_2_ at the up edge, a layer of carbon atoms with N doping, and C and H at the down edge, as shown in the inset of figure 6 a. They are obtained by summing their individual orbital PDOS contributions for each atom in the respective part^29^. We can see that the broad DOS peaks mainly origin from the PDOS peaks on the upper edge with H_2_ passivation, and the PDOS on the edge with H passivation, while the middle carbon atom layer has lower DOS peaks. As a result, the dihydrogenated edge states mainly contribute to the magnetism of system. Figure 6b demonstrate that the isosurface of spin density (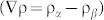) for M1, where the red and blue colors stand for the β-spin and α-spin components, respectively, and the isosurface level is taken as ±0.01|e|/Å^3^. The polarization in the A sublattice is much stronger than that in the B sublattice, and the spin direction of A and B sublattices is antiparallel. To our surprise, the stronger magnetism can be found at the dihydrogen, and a weak magnetism can be observed on the carbon atom with dihydrogen termination, while the atoms next to them feature a stronger spin polarization because it decays much more slowly to the center due to the different localization in the edge states^16^.

**Figure 8:**
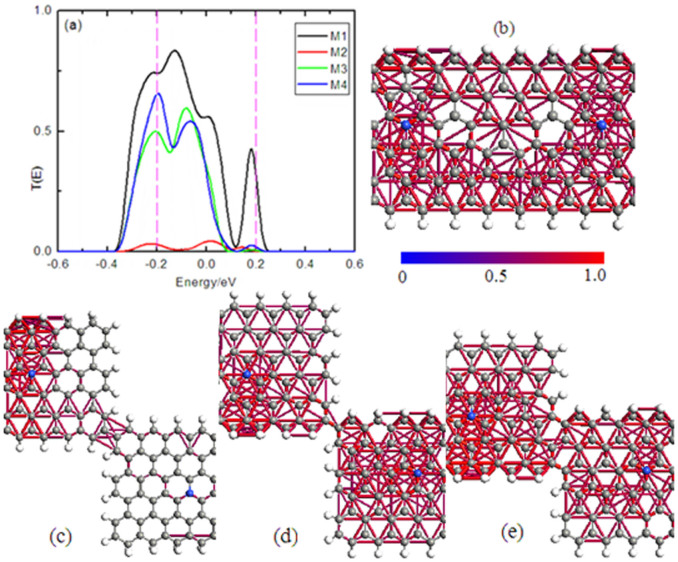
The transmission spectrum and local current. (a) The transmission spectrum for M1 ∼ M4 at 0.4 V bias, (b)–(e) the local β – spin currents at −0.2 eV energy with 0.4 V bias. The dashed lines denote the chemical potentials of left and right electrodes, and the Fermi level is set to zero.

To further understand the origin of these spin-resolved currents, we show the electron transmission spectrum (figure 8a), and transmission pathways (figure 8b ∼ 8e) and local currents (from the left to the right) of the β-spin for all models in the central region under 0.4 V bias at −0.2 eV energy. From figure 8a, we can see that the magnitude of transmission coeffcients within the bias windows show the following order: T_M1_ > T_M4_ > T_M3_ > T_M2_, leading to the obvious difference of currents upon the Landauer-Büttiker formula. Generally speaking, there exist two different current channels for electron transmission under bias: current via a chemical bond and through hopping between atoms of the same symmetry sub-lattice^24^,^30^. Neto et al proposed that electrons in graphene could hop between carbon atoms belonging to the same symmetry sublattice, but the hopping is very weak. However, our calculations show the hopping current is stronger, while bond current is very weak. More importantly, the hopping current show obviously stronger in the A sublattice as compared with that in the B sub-lattice. For the edge with the dihydrogen termination, the hopping currents emerge between carbon atoms with the same symmetry A sub-lattice, between A sublattice and B sub-lattice carbon atoms, also between dihydrogen atoms, as well as between dihydrogen atoms and A sub-lattice carbon. While for C–H bonded edge, the hopping currents happens between carbon atoms with the same symmetry A sub-lattice, and no hopping current between hydrogen atoms. As a result, a much stronger electron transmitting is displayed in the C–H_2_ bonded edge than that in the C–H bonded edge.

**Figure 9:**
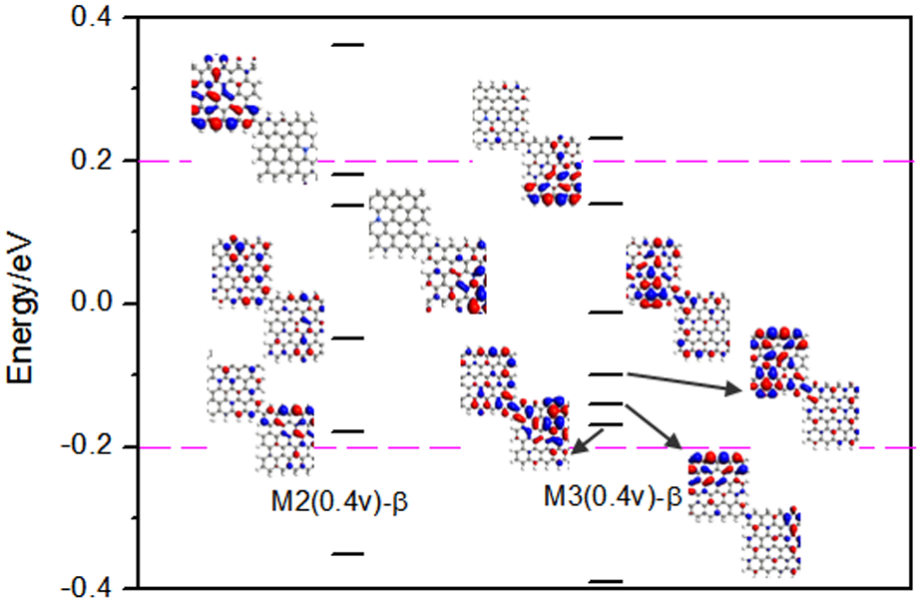
The β – spin Energy levels and some MPSH near Fermi energy. For molecular M2 and M3 at 0.4 V bias.

Moreover, the electronic tunneling and transport properties are intimately related to the molecular energy levels and their distribution in the energy space^31^,^32^,^33^. Energy levels of the β-spin state in the proximity of Fermi level and some molecular projected self-consistent Hamiltonian (MPSH) eigenstates for M2 and M3 at 0.4 V are exhibited in figure 9. Obviously, there are four energy levels for M2 within the bias window [−0.2, 0.2] eV, and the MPSH shows that these orbitals are localized on the right and left sides for the lowest unoccupied molecular orbital (LUMO) and LUMO + 1, and the highest occupied molecular orbital (HOMO) and HOMO-1 manifest that there exist some coupling between two linked C–H bonded edges. While for M3, there are five energy levels within the bias window, we can see that the LUMO, LUMO + 1, and LUMO + 3 levels show a good coupling between two linked C–H_2_ bonded edges, which are favorable for electronic tunneling and can make a larger contribution to the current. Therefore, the β-spin currents at 0.4 V for M3 will be larger than that for M2.

## Discussion

Based on the calculations by the non-equilibrium Green's function method combined with the density functional theory, we have systematically studied the N-doping position effects on the electronic structure, and find that the asymmetric ZGNRs (H_2_-6ZGNR-H) exhibit an interesting bipolar magnetic semiconducting behavior, which can be tuned to half-metals by nitrogen substitutions with only the β-spin channel conducting crossing the Fermi energy because the dopant atom N introduces an electron to the nanoribbon, which makes the Fermi level shift up to the conduction bands and cross with the bottom conduction band. Specially, the edge contact effects on the spin transport for the n-type doping H_2_-6ZGNR-H is investigated in details. The results show that a perfect spin filtering effect (100%) in such ZGNR nanojunctions can be achieved in a very large bias region for the unchanged spin states regardless of bias polarities, and the nanojunction with a contact of two C–H_2_ bonded edges has larger spin polarized current than that with a contact of two C–H bonded edges. demonstrate that the edge of C-H_2_ bonds play a crucial role for the spin magnetism and spin-dependent transport properties. Moreover, the negative differential resistance (NDR) effect is also observed in the spin-polarized current.

## Methods

In our studies, geometries optimization and calculations of electronic structure are performed by using the spin-polarized density functional theory (DFT)^34^,^35^. We employ Troullier-Martins norm-conserving pseudopotential to present the atom core and linear combinations of atom orbitals to expand the valence state of electrons. The local spin density approximation (LSDA) is used as the exchange-correlation functional. The equilibrium density matrix (DM) is evaluated by a contour integration method, while the nonequilibrium DM is numerically computed on a line with an imaginary part of 0.001 eV, which is parallel to real axis in complex plane. The real space grid techniques are used with the energy cutoff of 150 Ry as a required cutoff energy in numerical integrations and the solution of Poisson equation using fast fourier transform (FFT). The k-point sampling is 1, 1, and 500 in the x, y, z direction, respectively, where the z is the electronic transport one. Open boundary conditions are used to describe the electronic and the transport properties of nanojunctions. A vacuum layer of 15 Å is added to avoid the interaction between adjacent ribbons. The geometrical structures used are optimized until all residual forces on each atom are smaller than 0.05 eV/Å under the periodic boundary condition. The wave functions of C and H atoms are expanded by single-zeta polarized (SZP) basis set.

## Author Contributions

X.D. and Z.Z. performed the device design and theoretical analysis, G.T. and Z.F. calculated geometrical properties, electronic structures, the I-V characteristics, transmission spectra, and local currents, H.Z. and C.Y. studied the Energy levels and MPSH. All the authors discussed the results and wrote the manuscript.
